# Axial osteitis of the proximal sesamoid bones and desmitis of the intersesamoidean ligament in the hindlimb of Friesian horses: review of 12 cases (2002-2012) and post-mortem analysis of the bone-ligament interface

**DOI:** 10.1186/s12917-014-0272-x

**Published:** 2014-11-19

**Authors:** Harold Brommer, Margreet Voermans, Stefanie Veraa, Antoon JM van den Belt, Annette van der Toorn, Margreet Ploeg, Andrea Gröne, Willem Back

**Affiliations:** Department of Equine Sciences, Faculty of Veterinary Medicine, Utrecht University, P.O. Box 80.163, 3584 CM Utrecht, The Netherlands; Equine Veterinary Hospital Bodegraven, Bodegraven, The Netherlands; Equi-Tech, Puenta Piedra, Bogota, Columbia; Division of Diagnostic Imaging, Faculty of Veterinary Medicine, Utrecht University, Utrecht, The Netherlands; Biomedical MR Imaging and Spectroscopy Group, Image Sciences Institute, University Medical Center, Utrecht, The Netherlands; Department of Pathobiology, Faculty of Veterinary Medicine, Utrecht University, Utrecht, The Netherlands; Department of Surgery and Anaesthesiology, Faculty of Veterinary Medicine, Ghent University, Merelbeke, Belgium

**Keywords:** Hindlimb lameness, Clinical findings, Diagnostic imaging, Follow-up, Magnetic resonance imaging, Necropsy, Histopathology

## Abstract

**Background:**

Axial osteitis of the proximal sesamoid bones and desmitis of the intersesamoidean ligament has been described in Friesian horses as well as in other breeds. The objectives of this study were to review the outcome of clinical cases of this disease in Friesian horses and analyse the pathology of the bone-ligament interface. Case records of Friesian horses diagnosed with axial osteitis of the proximal sesamoid bones and desmitis of the intersesamoidean ligament in the period 2002-2012 were retrospectively evaluated. Post-mortem examination was performed on horses that were euthanized (n = 3) and included macroscopic necropsy (n = 3), high-field (9.4 Tesla) magnetic resonance imaging (n = 1) and histopathology (n = 2).

**Results:**

Twelve horses were included, aged 6.8 ± 2.7 years. The hindlimb was involved in all cases. Lameness was acute in onset and severe, with a mean duration of 1.9 ± 1.0 months. Three horses were euthanized after diagnosis; 9 horses underwent treatment. Two horses (22%) became sound for light riding purposes, 2 horses (22%) became pasture sound (comfortable at pasture, but not suitable for riding), 5 horses (56%) remained lame. In addition to bone resorption at the proximo-axial margin of the proximal sesamoid bones, magnetic resonance imaging and histopathology showed osteoporosis of the peripheral compact bone and spongious bone of the proximal sesamoid bones and chronic inflammation of the intersesamoidean ligament.

**Conclusions:**

Axial osteitis of the proximal sesamoid bones and desmitis of the intersesamoidean ligament in the hindlimb of Friesian horses carries a poor prognosis. Pathological characterization (inflammation, proximo-axial bone resorption and remodelling of the peripheral compact bone and spongious bone of the proximal sesamoid bones) may help in unravelling the aetiology of this disease.

## Background

Axial osteitis of the proximal sesamoid bones (PSBs) with desmitis of the intersesamoidean ligament (ISL) has been documented in several reports during the last two decades [1-8]. The clinical and diagnostic imaging features have been evaluated recently [7]. The disease is characterized by focal areas of bone lysis at the axial margin of the PSBs in combination with fraying and/or detachment of the ISL from the PSBs. The disorder is not new. A possible relationship between osteolytic changes of the PSBs and changes in the fibrillar structure of the ISL had already been hypothesized 80 years ago [9]. Causes of ISL desmitis that have been considered include primary disruption of the ISL [2,3], traumatically induced inflammation with secondary disruption of the ligament [2,3,7], disruption of the ISL secondary to sepsis of the metacarpophalangeal (MCPJ) or metatarsophalangeal joint (MTPJ) or digital flexor tendon sheath (DFTS) [2,5,8], fungal osteomyelitis of the PSBs [6], and ischemia-induced lysis of bone and secondary disruption of the ISL as a consequence of disturbance of the blood supply [1,2].

The architecture of the (micro-)vasculature of the PSBs had been reported to be of clinical relevance in those pathologies of the PSBs in which bone lysis is a predominant feature [10,11]. The vascular pattern of the PSBs and ISL is not essentially different between the medial and lateral PSBs and between fore- and hindlimbs: the arteries course through the bone in abaxial-to-axial, proximal-to-distal, and palmar-to-dorsal directions [10,11]. The vascularization of the ISL originates from a proximal branch of the sesamoid artery that arborizes into smaller branches in the ISL [10]. Traumatic disruption of the vessels or formation of vascular thrombosis may lead to ischemia-induced lysis of bone at the axial aspect of the PSBs at the level of the interface with the ISL [1,2].

The high number of Friesian horses (39%) in the study population of Vanderperren et al. [7] may suggest a relatively high susceptibility of the Friesian horse for development of axial osteitis of the PSBs with desmitis of the ISL. This study focuses entirely on this breed. Where diagnostic imaging (radiography (Rx), ultrasonography (US) and contrast enhanced computed tomography (CT)) was the central theme of the paper of Vanderperren et al. [7], the aim of the present study was firstly to review the outcome of Friesian horses diagnosed with and treated for axial osteitis of the PSBs with desmitis of the ISL, and secondly to describe the pathology of the bone-ligament interface. For the latter, low-field (0.27 Tesla (T)) and high-field (9.4 T) magnetic resonance (MR) imaging and histopathology were applied to a limited number of horses with this disease that became available for scientific research.

## Methods

### Case selection

Case records of Friesian horses admitted to the Department of Equine Sciences of Utrecht University (The Netherlands) between 2002-2012 (n = 7) and to the Equine Veterinary Hospital Bodegraven (The Netherlands) between 2009-2010 (n = 5) that were diagnosed with axial osteitis of the PSBs and desmitis of the ISL in the hindlimb were reviewed. Information obtained from these records included: age and gender of the horses, affected limb, duration and severity of lameness (graded according to the American Association of Equine Practitioners classification [12]), results of diagnostic tests and diagnostic imaging, treatments, and outcome (Table 1). Information of horses that were euthanized and subsequently subjected to post-mortem examination was also included.

**Table 1:** **Cases (n = 12) diagnosed with axial osteitis of the PSBs and desmitis of the ISL**

**Case**	**Age (years)**	**Gender**	**Affected limb, duration and severity of lameness**	**Diagnostic procedures**	**Therapeutic procedures/post-mortem examinations**	**Rehabilitation protocol**	**Follow-up**
1	8	Mare	Right hind 2 months 4/5	Clinical examination, perineural and intrasynovial anaesthesia, Rx, US	Arthroscopy and tenoscopy, NSAIDs for 2 weeks	Box rest/handwalking for 3 months	Persistently lame, euthanasia 6 months after treatment
2	9	Gelding	Left hind 2 months 4/5	Clinical examination, perineural and intrasynovial anaesthesia, Rx, US, CT, contrast enhanced CT, MR imaging (0.27 T), synovial fluid bacteriology and cytology of the MTPJ	No treatment, euthanasia, arthroscopy and tenoscopy post-mortem, macroscopic necropsy, histopathology	-	-
3	4	Gelding	Left hind 3 months 4/5	Clinical examination, perineural and intrasynovial anaesthesia, Rx	Arthroscopy and tenoscopy, NSAIDs for 1 week	Box rest/handwalking for 3 months, followed by pasture exercise	Sound for light riding purposes 4 months after treatment
4	3	Gelding	Left hind 1 month 3/5	Clinical examination, perineural anaesthesia, Rx, US, synovial bacteriology and cytology of the MTPJ	NSAIDs for 2 weeks	Box rest/handwalking for 3 months	Sound for light riding purposes 3 months after treatment
5	5	Gelding	Right hind 6 weeks 4/5	Clinical examination, perineural anaesthesia, Rx, US	NSAIDs for 2 weeks	Box rest/handwalking for 2 months	Similar lesion left hind after 2 months, euthanasia
6	5	Gelding	Left hind 2 months 3/5	Clinical examination, perineural anaesthesia, Rx, US	NSAIDs for 2 weeks, corticosteroids in MTPJ, orthopaedic shoeing	Pasture exercise	Pasture sound after 6 months
7	7	Gelding	Left hind 2 months 4/5	Clinical examination, perineural anaesthesia Rx, US	Arthroscopy and tenoscopy, NSAIDs for 3 weeks, corticosteroids in MTPJ, shockwave, orthopaedic shoeing	Pasture exercise	Intermittently lame up to 6 months after treatment
8	6	Mare	Right hind 6 weeks 3/5	Clinical examination, perineural and intrasynovial anaesthesia, Rx, US	Arthroscopy, NSAIDs for 2 weeks, shockwave	Box rest/handwalking for 3 months	Intermittently lame 3 months after treatment
9	6	Mare	Right hind 4 months 4/5	Clinical examination, perineural and intrasynovial anaesthesia, Rx, US, CT, contrast enhanced CT, MR imaging (0.27 T)	No treatment, euthanasia, post-mortem high-field MR imaging (9.4 T), macroscopic necropsy, histopathology	-	-
10	6	Stallion	Right hind 1 week 4/5	Clinical examination, Rx, US	Arthroscopy, platelet rich plasma in MTPJ, regional perfusion with tiludronate, NSAIDs for 5 weeks	Box rest/handwalking for 3 months, followed by pasture exercise	Persistently lame up to 6 months after treatment
11	10	Mare	Left hind 3 months 3-4/5	Clinical examination, perineural and intrasynovial anaesthesia, Rx, US	Tiluronate IV, NSAIDs for 2 weeks, shockwave	Box rest/handwalking for 1 month, followed by pasture exercise for 2 months	Pasture sound 3 months after treatment
12	13	Gelding	Right hind 4 weeks 3/5	Clinical examination, perineural and intrasynovial anaesthesia, Rx, US	No treatment, euthanasia, macroscopic necropsy	-	-

Demographic and clinical details, diagnostic procedures, therapeutic interventions/post-mortem examinations and follow-up (n = 12). Rx: radiography, US: ultrasound, CT: computed tomography, MR: Magnetic Resonance, NSAIDs: non steroidal anti inflammatory drugs, MTPJ: metatarsophalangeal joint.

### Animal care and ethics committee

The evaluated horses were all patients that were referred to the clinic and were examined and treated with informed consent of the owner. According to Dutch law there was no need for an Animal Care and Ethics Committee approval.

### Diagnostic procedures

Diagnostic procedures consisted of a clinical orthopaedic examination (n = 12) including diagnostic analgesia (n = 11) [13], clinical laboratory evaluation and bacteriological analysis of synovial fluid (n = 2) and various imaging modalities. Clinical examination was performed by diplomates of the European College of Veterinary Surgeons (ECVS, n = 4) or by a resident (n = 1) who was supervised by ECVS diplomates. The radiographic investigation of the MTPJ (n = 12) consisted of lateromedial, dorsal 5-10° proximal-plantarodistal oblique, dorsal 45° lateral-plantaromedial oblique, dorsal 45° medial-plantarolateral oblique, and high contrast dorsal 5-10° proximal-plantarodistal oblique views [14] using standard equipment^a^. Loss of radiopacity, which is consistent with loss of bone, was described in a subjective way in terms of ‘minor’, ‘moderate’ or ‘severe’. Decrease of radiopacity at the proximo-axial aspect was compared to the disto-axial aspect of the PSBs and was assessed in terms of ‘more’ or ‘equal’. US^b^ of the plantar aspect of the MTPJ (n = 11) was performed in the transverse and longitudinal planes with a 12 MHz linear transducer [15]. In the cases examined by CT^c^ (n = 2), contiguous slices of 3 mm thickness were acquired at settings of 120 kV and 220 mA with a rotation speed of 1 s [16]. For intra-arterial contrast enhanced CT of the MTPJ (n = 2) similar settings were used. Contrast medium^d^ (350 mg I/mL) was infused into the dorsal metatarsal artery using a remotely controllable infusion pump^e^ at a continuous rate of 2 mL/s [17]. Low-field (0.27 T) MR imaging^f^ (n = 2) was performed using techniques described by Werpy [18]. Table 2 details the MR imaging data. For arthroscopy of the MTPJ (n = 6, divided in 5 cases ante-mortem with subsequent surgical debridement and 1 case post-mortem) and tenoscopy of the DFTS (n = 4, divided in 3 cases ante-mortem with subsequent surgical debridement and 1 case post-mortem) the techniques described by McIlwraith et al. [19] and Nixon [20] were applied using standard instrumentation^g^. Not every case was subjected to all these procedures (Table 1).

**Table 2:** **Low-field (0.27 T) and high-field (9.4 T) MR imaging data**

	**TR (ms)**	**TE (ms)**	**TI (ms)**	**Flip angle (°)**	**Slice thickness (mm)**	**NEX**	**FOV (mm)**	**Matrix**	**Pixel size (μm)**
**Low-field (0.27 T)**									
Flash 3 dimensional T1 GRE	34	12		30	1.07	4	160 × 160 × 60	256 × 256 × 32	625
STIR	3200	48	90		4	5	180	256 × 256	703
T2 DSE	4080	106			3	2	160	256 × 256	625
Proton Density DSE	4080	26			3	2	160	256 × 256	625
**High-field (9.4 T)**									
3 Dimensional GRE	3.2	2.65		5		8	70 × 70 × 35	256 × 256 × 128	274
				10					
				20					
				40					
				60					
				90					

T: Tesla, TR: repetition time, TE: echo time, TI: inversion time, NEX: number of excitations, FOV: field of view, GRE: gradient echo, STIR: short tau inversion recovery, DSE: dual spin echo.

### Therapeutic procedures

Treatments consisted of one or more of the following options: systemic administration of non-steroidal anti inflammatory drugs (NSAIDs, n = 9) for 1-5 weeks (meloxicam^h^ 0.6 mg/kg bodyweight (bwt) Semel in Die (s.i.d.) per os (PO), flunixine meglumine^i^ 1.0 mg/kg bwt s.i.d./Bis in Die (b.i.d.) intravenously (IV), or phenylbutazone^j^ 2.2-4.4 mg/kg bwt s.i.d./b.i.d. PO), application of osteoclast inhibitors (sodium tiludronate^k^) by IV infusion (0.1 mg/kg bwt s.i.d. for 10 days, n = 1) or by regional perfusion (150 mg dissolved in 50 mL of Ringer’s solution, n = 1), intra-articular medication into the MTPJ (triamcinolone acetonide^l^ 10 mg (n = 2) or platelet rich plasma^m^ (n = 1)), orthopaedic shoeing (egg bar shoes or shoes with heel extensions, shoe changes every 6-8 weeks, n = 2), radial pressure wave therapy^n^ (3 times with 2 weeks interval, 2000 pressure waves, frequency of 15 Hz, maximal pressure of 4 N/cm^2^, n = 3), arthroscopic/tenoscopic surgery of the MTPJ (n = 5) and DFTS (n = 3) [19,20], and rehabilitation (stall confinement, handwalking or confined pasture exercise for 4-12 weeks, n = 9). Table 1 details the treatments in each case.

### Follow-up

Follow-up information consisted of repeated examination at the hospital or telephone enquiry with the owner 2-6 months after treatment. The time interval after initiation of treatment as well as degree of clinical lameness and level of performance were recorded.

### Post-mortem examination

Three cases were euthanized after the diagnosis of axial osteitis of the PSBs and desmitis of the ISL was made. In 1 case, diagnostic arthroscopy of the MTP and tenoscopy of the DFTS was performed after euthanasia. Macroscopic necropsy of the lame limb was performed in 3 cases and a comparison was made with the ISL-PSB interface of a non-diseased horse that had been euthanized for reasons unrelated to the musculoskeletal apparatus.

Histopathological examination was performed in 2 cases and in a non-diseased Friesian horse euthanized for reasons unrelated to the musculoskeletal apparatus. Formalin-fixed, decalcified, paraffin-embedded, and haematoxylin and eosin-stained slices with a thickness of 3 μm were produced of the interface between the PSBs and the ISL.

### High-field MR imaging

High-field (9.4 T) MR imaging° was performed in one isolated and formalin-fixed specimen of the PSBs and ISL of a diseased Friesian horse and of a specimen of a non-diseased adult Friesian horse, euthanized for reasons unrelated to the musculoskeletal apparatus. The specimens were placed in a quadrature volume coil; Table 2 further details the MR imaging data. Data were stored in neuroimaging informatics technology initiative (NIfTI) format and were read with image analysis software^p^.

## Results

### Case details

Twelve cases of axial osteitis of the PSBs with desmitis of the ISL were included in the study (Table 1). The mean age was 6.8 ± 2.7 years (range 3-13 years). All horses had a history of intermittent severe hindlimb lameness of an acute onset with a mean duration at the time of orthopaedic examination of 1.9 ± 1.0 months (range 1 week - 4 months). No treatments prior to referral other than administration of NSAIDs and a period of box-rest had been applied by the owners or referring veterinarians with no or minor improvement of the lameness.

### Clinical findings

At the time of examination, the horses displayed a lameness of 3-4/5. The right hindlimb and left hindlimb were affected in an equal number of 6 cases. All horses resented normal loading of the fetlock and there was reduced extension of the fetlock at walk, especially on the circle when the affected limb was inside. All horses had effusion of the MTPJ (the plantar pouch being more obviously distended than the dorsal pouch) and a marginal to moderate effusion of the DFTS. Pain could not be evoked by palpation or by passive movement of the distal limb. Distal limb flexion increased the lameness. Plantar nerve blocks (n = 11) performed at the base of the PSBs did not result in any substantial improvement of the lameness. However, there was 60% to 80% improvement after low 6 point nerve blocks (perineural anaesthesia of the medial and lateral plantar nerves, medial and lateral plantar metatarsal nerves and medial and lateral dorsal metatarsal nerves at a level just proximal to the MTPJ, n = 11). After intra-articular anaesthesia of the MTPJ (using 8-10 mL of local anaesthetic solution, n = 7), a mean improvement of 40-60% was noted after 10 minutes. Intrathecal anaesthesia of the DFTS (using 6-8 mL of local anaesthetic solution, n = 4) also resulted in a mean improvement of 40-60% 10 minutes after injection. The synovial fluid was yellow and clear at inspection, viscosity appeared to be slightly reduced, mean polymorphonucleated cell count was 4.0 ± 1.0 × 10^9^ cells/L and mean total protein concentration was 1.2 ± 0.2 g/dL (n = 2). Culture of the synovial fluid for bacterial or fungal organisms was negative in both of the tested samples (n = 2).

### Findings on diagnostic imaging

Characteristic radiographic changes could be discerned on the (high contrast) dorsal 5-10° proximal-plantarodistal oblique views of the MTPJ. Loss of radiopacity of variable extent from minor to severe was present at the proximo-axial aspects of the PSBs, symmetrically medially versus laterally. Loss of radiopacity was more extensive at the proximo-axial and less extensive at the disto-axial aspects of the PSBs. The margins of the areas where loss of radiopacity was noted, were not well defined.

US showed diffuse and irregular hypoechogenicity of the ISL on transverse US images. The interface between the ISL and the axial compact bone of the PSBs was irregular in all cases in which US was performed (n = 11). In 3 cases small hyperechogenic particles were detected at the interface between compact bone and ligament at the proximo-axial aspect of the PSBs, which was most likely consistent with avulsion fragments.

On pre-contrast CT images (n = 2) ill-defined osteolytic areas at the proximal aspect of the axial border of the PSBs were detected. The area of bone lysis was more or less symmetrical medially and laterally with respect to size and location. Intra-arterial contrast CT imaging (n = 2) revealed a focal deposit of contrast medium at the level of the proximal part of the ISL, which was consistent with either abnormal blood vessel permeability/disruption or neovascularization related with tissue repair.

**Figure 1:**
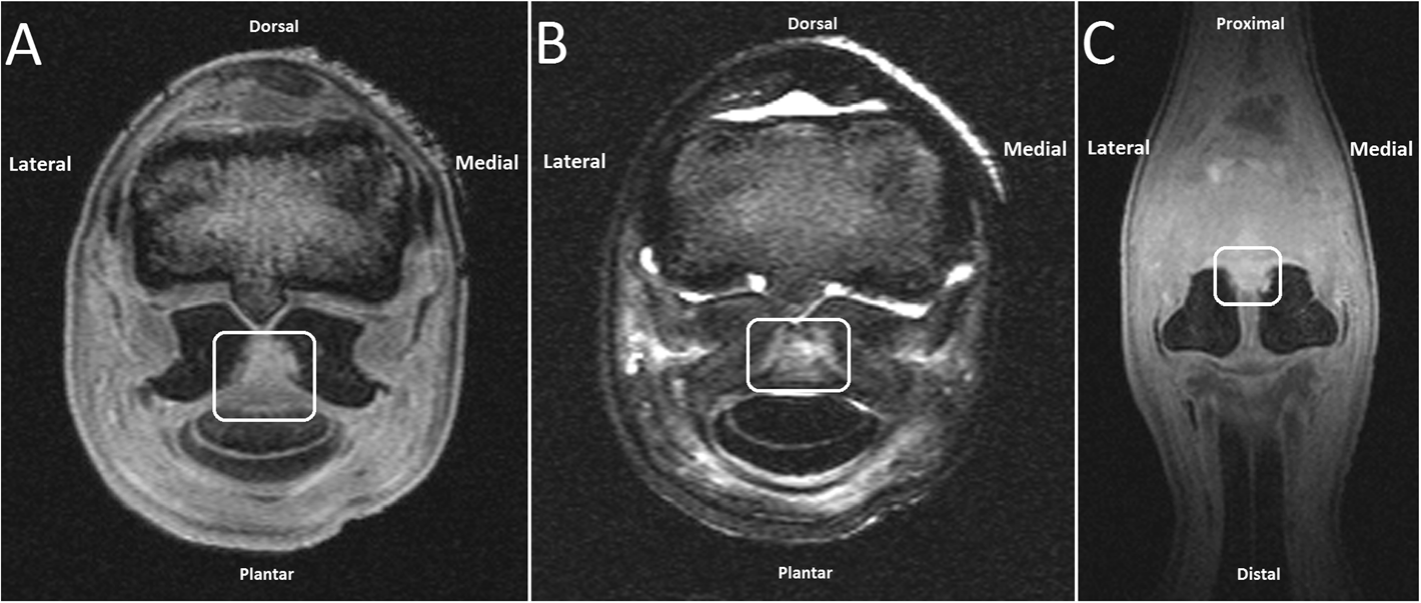
**Low-field (0.27 T) MR imaging of the MTPJ. A)** T1 weighted GRE transverse image at the level of the PSBs and ISL. **B)** STIR transverse image at the same level. **C)** T1 weighted GRE dorsal image. The proximal part of the ISL shows an iso-intense signal compared to surrounding tissues on T1 weighted GRE images (A, C, white line marked area) and an irregular hyperintense signal on STIR images (B, white line marked area). A slightly ill-defined increase in signal intensity was also present at the proximo-axial aspect of the compact bone of the PSBs and extending slightly into the spongiosa on all sequences. The margins of the compact bone are irregular. Note the diffuse hypointense signal of the PSBs on the T1-weighted images (A, C). These findings may be consistent with inflammation and fibrous tissue (scar) formation in the ligament and adjacent region, loss of compact bone at the proximo-axial aspect and edema in the adjacent spongious bone of the PSBs.

On low-field MR imaging (n = 2), the proximal part of the ISL had an iso-intense signal (compared to surrounding tissues) on T1-weighted gradient echo (GRE) images and an irregular hyperintense signal on proton density, T2-weighted and short tau inversion recovery (STIR) images. These findings may be consistent with inflammation and fibrous tissue (scar) formation in the ligament and adjacent region. On all sequences, a slightly ill-defined increase in signal intensity was also present at the proximo-axial region of the PSBs, the region that shows loss of radiopacity on radiographs and that normally consists of compact bone. At these sites, the compact bone did not have a regular margin and there was no definite interface with adjacent spongious bone. Adjacent spongious bone of the PSBs had a diffuse hypointense signal intensity on T1-weighted GRE images and a slightly diffuse hyperintense signal on proton density, T2-weighted and STIR images; taken all together these findings are suggestive for loss of compact bone at the proximo-axial region and edema whether or not accompanied by necrosis in the adjacent spongious bone of the PSBs (Figure 1).

During arthroscopy of the MTPJ and tenoscopy of the DFTS, mild hypertrophy of the synovial membrane was visible. In the MTPJ, discolouration and fraying of the ISL was found in 3 cases. Complete tearing of the ISL with penetration into the DFTS was present in 2 cases. In the other case, the plantar surface of the ISL was not affected. At the level of insertion of the ISL onto the PSBs, the articular cartilage was soft at manual probing. Debridement of malacic bone using hooked curettes and motorized equipment^g^ was performed until healthy subchondral bone was encountered. Torn fibres of the ISL were also debrided. Debridement was performed by either a MTPJ approach in cases in which the dorsal surface of the ISL was affected but not the plantar surface, or by a combined MTPJ and DFTS approach in the cases in which complete tearing of the ISL into the DFTS was present. We had no cases with incomplete tearing of the plantar surface of the ISL in combination with an intact dorsal surface. There were no changes in the superficial digital flexor tendon, deep digital flexor tendon, proximal and distal manica flexoria, or the proximal annular ligament in any of the cases.

### Follow-up

Nine horses were subjected to treatment (Table 1). Two horses (22%) became sound for light riding purposes 3-4 months after treatment. Two horses (22%) were pasture sound 3-6 months after treatment but were not used for riding. Five horses (56%) remained intermittently or persistently lame. One of these developed axial osteitis of the PSBs with desmitis of the ISL in the contralateral hindlimb after 2 months and was subsequently euthanized. One other horse was euthanized 6 months after treatment due to persistent lameness.

### Post-mortem examination

**Figure 2:**
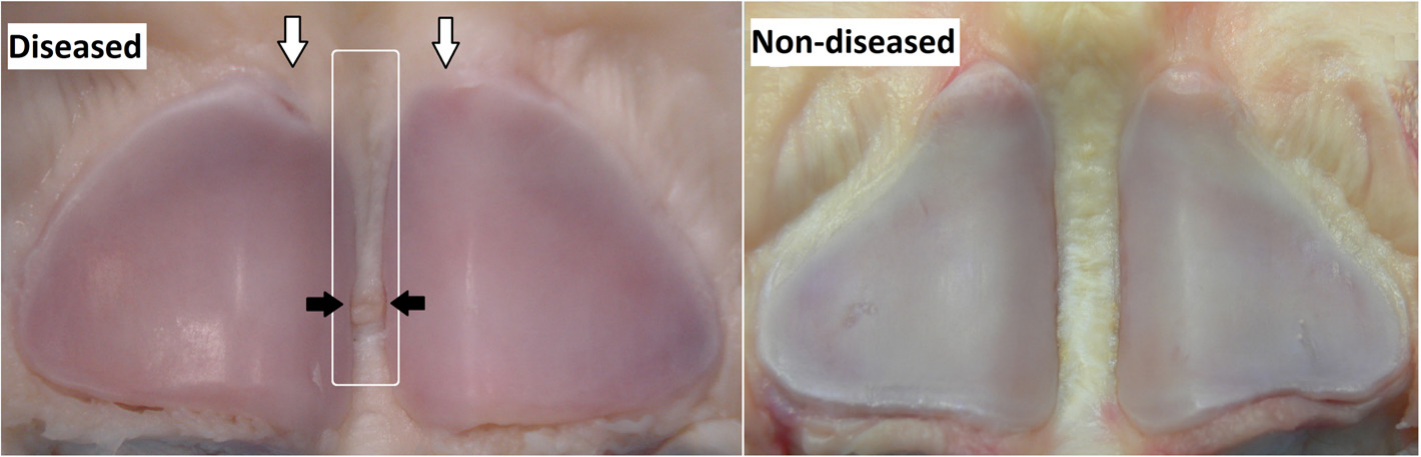
**Macroscopic view of the PSBs and the ISL of a diseased and a non-diseased Friesian horse.** Note the focal loss of bone at the proximo-axial aspects of the PSBs (open arrows). Concurrent partial rupture of the ISL (white line marked area), starting at the proximal level of the bone and coursing to distal with locally a detachment from the bone (black arrows). The rupture did not enter the plantar surface so there was no penetration into the DFTS in this case.

Three horses were euthanized on request of the owner after the diagnosis of axial osteitis of the PSBs with desmitis of the ISL and were subjected to post-mortem examination. Macroscopic inspection showed a discoloured, irregular and frayed ISL. Focal loss of bone was present at the proximo-axial aspects of the PSBs. The attachment of the ISL to the bone was irregular with focal detachment of the ISL (Figure 2).

**Figure 3:**
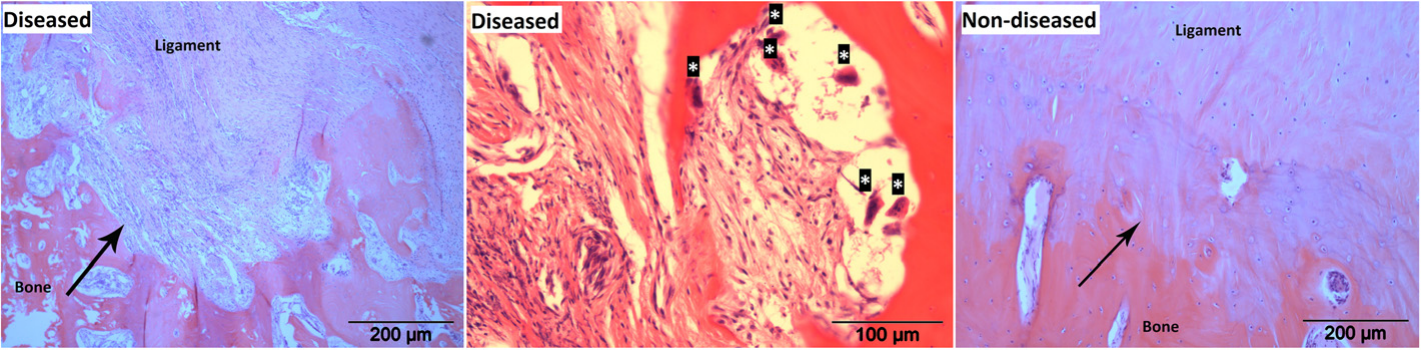
**Histopathology of the interface of the PSBs and the ISL.** Transverse sections of a diseased and a non-diseased Friesian horse, haematoxylin and eosin staining. Note the multifocal to coalescing inflammation of the ISL which is characterized by abundant fibroblasts, lymphocytes and plasma cells in the diseased horse. The transition from ligament to bone (arrow) is very irregular in the diseased horse and compared to the non-diseased horse, there is a decrease of surface area where ligament tissue merges to bone in the diseased horse. Remaining adjacent bone shows increased osteoclastic bone resorption (osteoclasts marked with asterisks). Bone marrow was hypercellular due to invasion of lymphocytes and plasma cells.

At histopathology, severe multifocal to coalescing areas of inflammation of the ISL, characterized by abundance of fibroblasts, lymphocytes, plasma cells and a small amount of necrotic debris were visible in the specimens of the diseased horse. The transition from ligament to the compact bone was very irregular. In addition, compared to the non-diseased horse, in the specimens of the diseased horse there was a decrease of available surface at which ligament tissue could merge to the compact bone due to loss of bone at the bone-ligament interface. Remaining adjacent bone showed increased osteoclastic bone resorption when comparing the diseased horse to the non-diseased horse. Bone marrow was hypercellular due to an invasion of lymphocytes and plasma cells. These histological abnormalities were found along the entire line of attachment of the ISL to the PSBs and were most severe in the medial PSB (Figure 3).

### High-field MR imaging

**Figure 4:**
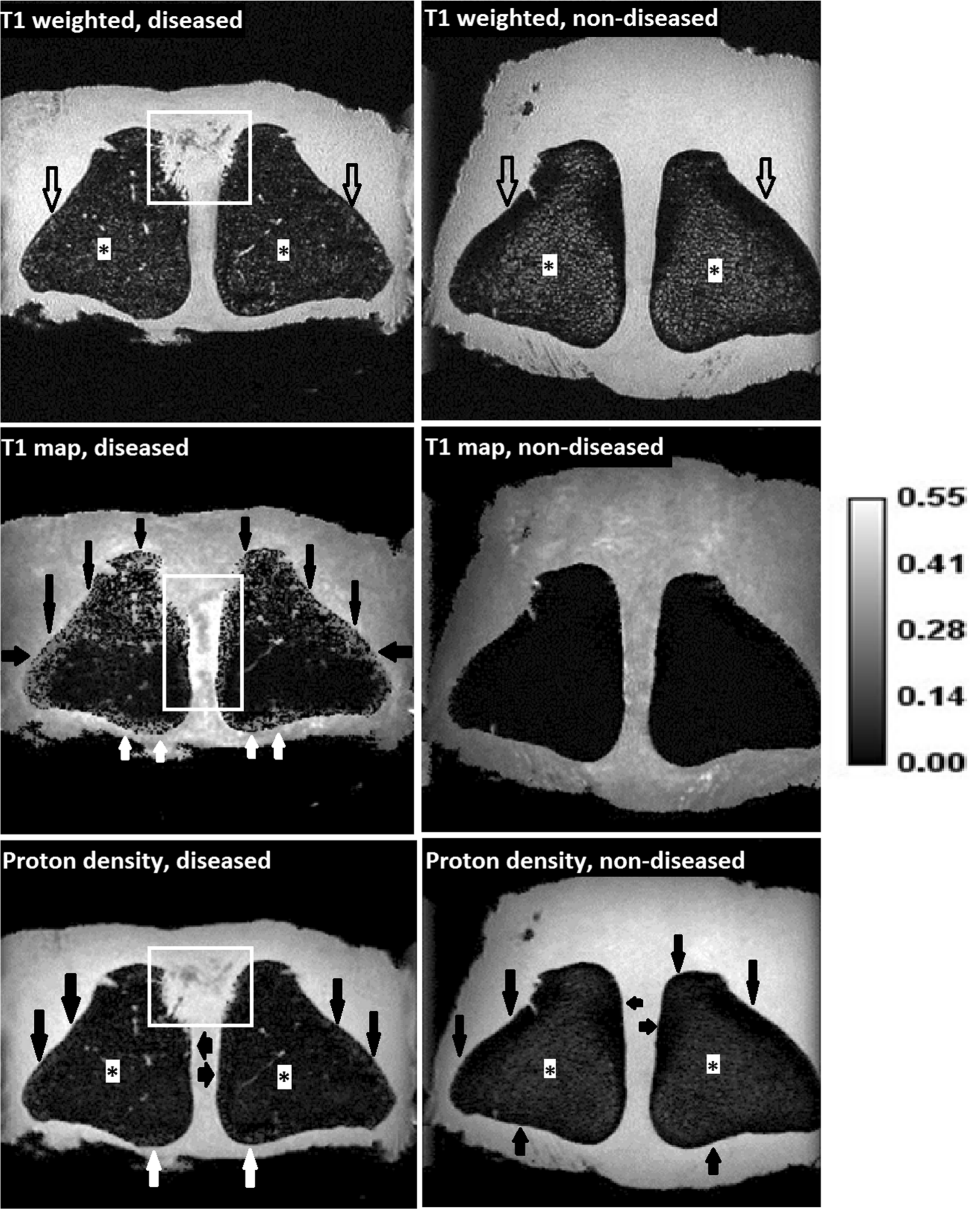
**High-field MR imaging (9.4 T, flip angle 40°) of the PSBs and the ISL.** Post-mortem analysis of a specimen of a Friesian horse with axial osteitis of the PSBs and desmitis of the ISL (left images) and a non-diseased Friesian horse (right images). Top: T1 weighted GRE dorsal sequences. Middle: T1 mapping. Bottom: proton density sequences. On the T1 weighted images, a clear increase in signal intensity could be seen in the compact bone at the proximo-axial area of the PSBs in the diseased horse (white line marked area). Compared to the non-diseased horse, signal intensity of the spongiosa (asterisks) was reduced, signal intensity of the compacta was increased in the T1 weighted GRE (open arrows) and proton density (black arrows) images leading to homogeneous signal intensity across the PSBs in the diseased animal. In the non-diseased horse, heterogeneous signal intensity was present in T1 weighted and proton density images with the spongiosa having more intense signaling (asterisks) and peripheral compacta having less intense signaling (open arrows). Compared to the non-diseased horse, on the T1 map of the diseased horse, especially the apical part and the peripheral compact bone showed an increase in T1 values (black and white arrows), the ISL ligament also showed an increase in T1 values (white line marked area). The non-diseased horse showed a homogeneous pattern of the PSBs with hardly any signal on the T1 map. Integration of the findings on all images could be interpreted as loss of compact bone at the proximo-axial margin of the PSBs, osteoporosis of the peripheral compact bone and spongious bone of the PSBs, and inflammation and fibrous (scar) tissue formation of the ISL in the diseased horse.

Compared to the non-diseased horse, a clear increase in signal intensity could be seen in the compact bone at the proximo-axial margin of the PSBs on the T1-weighted images of the diseased horse (Figure 4). The interface with the spongious bone at the proximo-axial region is irregular and ill-defined. Moreover, a diffuse hypointense signal without difference in signal intensity between the central spongious bone and the peripheral compact bone was present on T1-weighted GRE images of the diseased horse. The signal intensity was more or less homogeneously distributed across the spongiosa and compacta. In the non-diseased horse, signal intensity of the PSBs was heterogeneous with the central spongious bone having more intense signals compared to the peripheral compact bone. Evaluating the absolute T1 values (T1 map) of the diseased horse, especially the proximal (apical) part and the peripheral compact bone showed local increases of T1 values compared to the non-diseased horse, leading to a heterogeneously distributed T1 pattern across the PSBs of the diseased horse. Moreover, the ISL ligament of the diseased horse showed higher T1 values than the non-diseased horse on the T1 map. The T1 map of the non-diseased horse showed a more homogeneous pattern across spongiosa and compacta of the PSBs with hardly any signal. Proton density images of the diseased horse had a clear increase in signal intensity of the compact bone at the proximo-axial margin of the PSBs compared to the images of the non-diseased horse. The remainder cortical and spongious bone of the PSBs showed a homogeneous distribution of signal intensity in the diseased horse. In the non-diseased horse there was a more heterogeneous signal intensity pattern across the PSBs, the signal of the central spongiosa was slightly more intense compared to the peripheral compacta. Taking the findings of all images together, the results are consistent with loss of compact bone at the proximo-axial margin of the PSBs, osteoporosis of the peripheral compact bone and spongious bone of the PSBs and inflammation and fibrous (scar) tissue formation of the ISL in the diseased horse.

## Discussion

The clinical profile and pathologic findings of these cases of axial osteitis of the PSBs with desmitis of the ISL in Friesian horses showed a consistent pattern. The disease was typically diagnosed in young to middle-aged Friesian horses and characterized by acute hindlimb lameness localised in the MTPJ. Findings on Rx, US and (contrast enhanced) CT matched closely with those previously reported [1,2,6-8]. The overall prognosis in this case series was poor with return to light riding purposes as the best achieved result. The majority of the horses remained lame and, depending on severity of the lameness, became pasture sound or had to be euthanized.

It should be realized that retrospective evaluation of data carries limitations, especially when it is used to answer questions about outcome in terms of relative performance after treatment. Further, the highly heterogenic character of the treatment regimens to which the horses were subjected precludes drawing of any evidence-based conclusions regarding treatment efficacy, which is a weakness of this study. Treatments of the horses in the study of Dabareiner et al. [2] were more uniform and they reported better results in a case series involving 4 Quarter Horses, 2 Polo Ponies, 1 Thoroughbred and 1 Appaloosa. In that study five MTPJs and three MCPJs were treated with a combination of surgical intervention (arthroscopic/tenoscopic debridement) and medical treatment and an overall result of 63% return to previous level of performance was reported [2]. Although the horses in that study differ from Friesian horses in several aspects such as conformation and equestrian use, the results may suggest that aggressive treatment in the form of arthroscopic/tenoscopic debridement of bone and inflamed ligament tissue may optimize the conditions for healing. In our opinion, if the plantar surface of the ISL (i.c. at the site of the DFTS) looked unaffected, care should be taken not to transect the ISL as this may lead to instability of the MTPJ.

Another factor that may influence outcome is the duration of lameness before treatment. In our case series, the mean lameness duration before referral was 1.9 months. In the case series of Wisner et al. [8], who also reported a poor outcome, lameness duration was still longer with a mean of 5.6 months. In the study of Dabareiner et al. [2], which reported better results, mean lameness duration was only 3.1 weeks. This may indicate that, as with many disorders, the sooner treatment is started, the better the prognosis.

Some of the horses described by Dabareiner et al. [2] had an infective component which was a negative prognostic indicator in that study and in other studies reported a few years later [1,6]. Infection should, however, be regarded as a separate condition. In our cases, there were no histories or signs of infection.

The rationale for the use of corticosteroids in some of our cases was its anti-inflammatory effect and reduction of joint/sheath effusion which may lead to reduction of the lameness. However, corticosteroids also have anti-anabolic action which is an undesired side-effect in damaged tissue. It is therefore necessary to weigh up carefully how corticosteroids should be used in this disease.

In one case axial osteitis of the PSBs with desmitis of the ISL had been developed in the contralateral hindlimb after 2 months of rehabilitation. We usually advise owners to radiograph the limbs bilaterally at initial examination. In this case, the owner refused to radiograph the contra-lateral MTPJ for financial reasons. In every case and at every time, the cost-benefit ratio should be considered. From a prognostic point of view this case showed the importance for bilateral imaging and this is a valid argumentation of acquisition of at least a dorsoplantar radiograph of the contralateral limb.

The cost-benefit ratio should also be discussed in every clinical case when considering advanced diagnostic imaging such as (contrast enhanced) CT of MR imaging versus surgical arthroscopy/tenoscopy. Advanced diagnostic imaging techniques as (contrast enhanced) CT and MR imaging as used in this paper have provided in further documentation of this disease, but we believe that for routine clinical practice these techniques have very little additional value over Rx and US for differentiation between different treatment options or for defining the prognosis. As we believe that surgical debridement is likely to give the best outcome, we would advise the owner to invest his/her money for surgery rather than for advanced diagnostic imaging in cases in which the owner opts for treatment.

It has been speculated that excessive or abnormal forces within the MTPJ, provoked by severe overextension of the joint in combination with hindlimb rotation in particular, will heavily load the plantar supporting structures, especially the PSBs and the ISL [2]. Friesian horses might be predisposed for axial osteitis of the PSBs with desmitis of the ISL. Tendon mechanical properties in this breed have been shown to be on average more compliant than in ponies, resulting in hyperextension of the MTPJ [21]. Fetlock angles were not measured in this study because any increase in hyperextension due to different material properties would potentially be masked by decreased limb loading, related to the grade of lameness.

In discussions on the aetiopathogenesis of this disorder, it is debatable whether ligament pathology comes first with bone pathology as a secondary event, or the other way round. It is also a matter of debate whether trauma is the primary initiating factor that evokes a cascade of secondary events consisting of inflammation, weakening/rupture of the ISL, disturbance of the blood supply of the bone-ligament interface and bone resorption. Another possibility is that disturbance of the blood supply due to vessel rupture or thrombosis formation is primary and bone lysis with inflammation of the ISL secondary. The information in the current paper does not permit to draw conclusions on these hypothesised pathophysiological sequences. In this sense this study was limited as macroscopic necropsy and histopathology were restricted to the lame limb. Concurrent post-mortem examination of the contralateral non-lame limb might possibly have resulted in additional information that may have assisted in unravelling the aetiopathogenesis of this disease.

The nature of the bone and the type of injury may play a role in the type of response that develops after tearing and avulsion of tendoligamentous insertions [22]. New bone formation as well as osseous cyst-like lesions have been reported in cases of desmopathy of collateral ligaments of the distal interphalangeal joint [23]. In axial osteitis of the PSBs with desmitis of the ISL bone lysis was the predominant feature of bone pathology. Bone lysis was seen at the axial aspect of the PSBs and more lysis was seen at the proximo-axial margin compared to the disto-axial aspect of the PSBs. The same distribution pattern of bone lysis was reported by Vanderperren et al. [7]. Dabareiner et al. [2], Sedrish et al. [5] and Sherman et al. [6] found relatively more bone lysis in the midaxial region of the PSBs, whereas Barr et al. [1] observed more bone lysis in the disto-axial region of the PSBs.

Given the vascularization pattern of the PSBs and ISL [10,11], an explanation for the typical bone resorption at the proximo-axial location could be the different density of afferent arterioles per unit of bone volume. Trumble et al. [11] observed a relative ‘absence of vessels’ in the apical region of the PSB in contrast to the rich vascularity in the remainder of the bone. Trauma to blood vessels or thrombosis formation will lead to changes in perfusion and disturbances in oxygen and nutrient supply at the apical region of the PSB. Intra-arterial contrast CT showed an increased density of vasculature in the region of the ISL, comparable to the findings described by Vanderperren et al. [7]. This indicates that the vascular pattern had changed in diseased horses, supporting the hypothesis that changes in the blood supply may play a role, either primary or secondary, in the development of this disease.

In addition to loss of bone at the proximo-axial margin of the PSBs, high-field MR imaging has learnt us that the remainder part of the PSBs also responds in this disease. High field MR imaging showed changes that could be interpreted as bone remodelling (development of osteoporosis) which was evident in the compact bone at the peripheral margins and the spongious bone of the PSBs. It remains elusive whether these phenomena are hallmarks of the primary disease process or represent disuse osteopenia which is a physiological adaptive response as a consequence of Wolff’s law [24] caused by the fact that the horse had been lame during a period of time.

## Conclusions

Axial osteitis of the PSBs and desmitis of the ISL should be considered in the differential diagnosis of severe hindlimb fetlock lameness in Friesian horses. Affected horses typically showed clinical signs of acute onset. The results of currently used treatments were disappointing. High-field MR imaging and histopathology provided evidence of interrelated changes of bone lysis at the axial aspect of the PSBs, inflammation with subsequent fibrous tissue (scar) formation of the adjacent ISL and additional remodelling (development of osteoporosis) of the compact bone at the periphery and the spongious bone of the PSBs. Further studies are needed to unravel the exact aetiopathogenesis of this disorder.

## Endnotes

^a^Philips Healthcare, Eindhoven, The Netherlands or Agfa Health Care Nederland B.V, Rijswijk, The Netherlands.

^b^Philips Healthcare, Eindhoven, The Netherlands or Aloka®, Biomedic Nederland B.V., Almere, The Netherlands.

^c^Philips Healthcare, Eindhoven, The Netherlands.

^d^Xenetix®, Guerbet B.V., Gorinchem, The Netherlands

^e^Mark V plus®, Medrad Co., Warrendale, USA.

^f^Siemens Healthcare Solutions Diagnostics, Breda, The Netherlands.

^g^KARL STORZ GmbH & Co. KG, Tuttlingen, Germany.

^h^Metacam®, Boehringer Ingelheim Vetmedica GmbH, Ingelheim am Rhein, Germany.

^i^Fynadyne®, Intervet International B.V., Boxmeer, The Netherlands.

^j^ProDynam®, Dechra Veterinary Products A/S, Uldum, Denmark.

^k^Tildren®, CEVA Sante Animale, Libourne, France.

^l^Kenacort®, Bristol-Myers Squibb B.V., Woerden, The Netherlands.

^m^Recover®, Biomet Biologics Inc., Warsaw Indiana.

^n^DolorClast®, Enraf-Nonius N.V., Aartselaar, Belgium.

^o^Horizontal bore MRI®, Varian Inc., Palo Alto, USA.

^p^ImageJ®, National Institute of Heath (NIH), Maryland, USA.

## Abbreviations

b.i.d: Bis in Die (Latin, twice a day)
bwt: bodyweight
CT: Computed tomography
DFTS: Digital flexor tendon sheath
DSE: Dual spin echo
ECVS: European College of Veterinary Surgeons
FOV: Field of view
GRE: Gradient echo
ISL: Intersesamoidean ligament
IV: Intravenously
MR: Magnetic resonance
MCPJ: Metacarpophalangeal joint
MTPJ: Metatarsophalangeal joint
NEX: Number of excitations
NIfTI: Neuroimaging informatics technology initiative
NSAIDs: Non steroidal anti inflammatory drugs
PO: Per os
PSBs: Proximal sesamoid bones
Rx: Radiography
s.i.d: Semel in Die (Latin, once a day)
T: Tesla
TE: Echo time
TI: Inversion time
TR: Repetition time
US: Ultrasonography
STIR: Short tau inversion recovery
